# A stronger association of diabetes mellitus with impaired hyperaemia using a novel ECG-gated device compared with peripheral arterial tonometry

**DOI:** 10.1007/s10792-022-02276-8

**Published:** 2022-06-07

**Authors:** Anchal Lal, Neha Dave, Michael Anthony Barry, Annika Sood, Paul Mitchell, Aravinda Thiagalingam

**Affiliations:** 1 Department of Cardiology, Westmead Hospital, Sydney, NSW Australia 2145; 2 Sydney Medical School (Westmead Clinical School), The University of Sydney, Sydney, NSW Australia 2145; 3 Centre for Vision Research, Westmead Institute for Medical Research, Sydney, NSW Australia 2145; 4 School of Medicine and Public Health, The University of Newcastle, Callaghan, NSW Australia 2308; 5 Department of Cardiology, Room 2082, Level 2, Clinical Sciences Corridor, Westmead Hospital, Cnr Darcy and Hawkesbury Roads, Westmead, Sydney, NSW Australia 2145

**Keywords:** Electrocardiogram, Diabetes mellitus, Hyperaemia, Retina, Tonometry, Vasodilation

## Abstract

**Purpose:**

Impaired digital reactive hyperaemia and flicker-stimulated retinal vascular response are commonly reported risk markers of cardiovascular disease. This is the first study to determine the correlation of these risk markers with diabetes mellitus by comparing our novel flicker-modulated ECG-gated fundoscope with the EndoPAT2000 system.

**Methods:**

In total, 119 controls and 120 participants with diabetes mellitus partook in this cross-sectional study. The EndoPAT2000 system assessed digital reactive hyperaemia under fasting conditions. A mydriatic ECG-gated fundoscope with a novel flicker module acquired digital retinal images of the left eye before, during and after flicker stimulation. An inhouse semi-automated software measured retinal vessel diameters using a validated protocol with two observers repeating measurements in a subset of 10 controls and 10 participants with diabetes mellitus. Intra- and inter-observer reliability analyses occurred by the interclass correlation coefficient. A receiver operating characteristic curve established associations of variables with diabetes mellitus.

**Results:**

Diabetes mellitus was more strongly associated with flicker-stimulated retinal arteriolar calibre change from baseline (AUC 0.81, 95% CI 0.75–0.87, *p* < 0.0001) than reactive hyperaemia index. Median flicker-stimulated arteriolar calibre change from baseline (controls: 2.74%, IQR 1.07 vs diabetes mellitus: 1.64%, IQR 1.25, *p* < 0.0001) and reactive hyperaemia index (controls: 1.87, IQR 0.81 vs diabetes mellitus: 1.60, IQR 0.81, *p* = 0.003) were lower in diabetes mellitus than controls. Intra- and inter-observer reliability coefficients were high from 0.87 to 0.93.

**Conclusions:**

Impaired flicker-stimulated retinal arteriolar calibre change from baseline is more highly correlated with diabetes mellitus in this study than a reduced reactive hyperaemia index.

## Introduction

Endothelial dysfunction is one of the earliest risk markers in diabetes mellitus and has an important role in microvascular changes associated with cardiovascular disease [1].hile there There are non-invasive endothelial function tests available, such as flow-mediated dilation and digital arterial tonometry. These have been validated and are highly reproducible in ideal circumstances, however, they pose several disadvantages. Flow-mediated dilation requires a highly skilled technician to assess accurate changes in the brachial artery lumen due to ischaemia [2]. Autonomic input is difficult to control in both procedures and can influence results [3], making these methods unreliable and unsuitable to implement clinically.

Retinal arterioles are free of autonomic stimulation and employ local autoregulatory mechanisms to adapt to the metabolic demands of tissues. Flicker stimulation to the eye causes an increased turnover of photoreceptors, thereby increasing oxygen demand [4] and blood flow to the retina [5]. Furthermore, retinal blood flow is higher with exposure to flickering light than constant light [6], which makes it easier to detect small changes in microvascular reactivity. All of these factors permit a properly equipped fundoscope to conduct direct non-invasive assessments of the human microvasculature for autoregulatory responses.

Flicker stimulation to the retina results in the local release of endothelial nitric oxide into the retinal microvasculature; the impairment of this response is linked with reduced endothelial nitric oxide synthase and nitric oxide production [4]. This property enables flicker-stimulated vasodilation to be useful in the investigation of endothelial function. Flicker-stimulated vasodilation does not require additional training of the operator nor a secluded room to reduce autonomic disturbance. It is therefore a convenient and safer alternative to hypercapnia- and hypoxia-induced retinal vasodilation.

Additionally, imaging assessment of retinal vessels is a well-recognised and validated method of exploring the retinal microcirculation, independently of the choroidal system [7]. Microvascular changes precede diabetes-related macrovascular complications and hence can be used to detect endothelial dysfunction earlier, before macrovascular changes occur. In diabetes mellitus, this is reflected by an early impaired flicker-stimulated retinal vascular response, [8] along with ischaemic and neural damage in the inner retina before the onset of clinically detectable diabetic retinopathy [9]. Abnormal flicker-stimulated retinal vessel calibre reactivity is also an important indicator of diabetic retinopathy progression.

We previously demonstrated that capturing photographs of the retina at the end of diastole (trough of the aortic pressure wave) improves the precision and reproducibility of the measurement of retinal vessel calibre, in both controls and diabetes mellitus [10]. ECG-gated retinal images at the end of diastole have also been captured in studies investigating flicker-stimulated retinal vasodilation as it enables detection of small changes in retinal vessel calibre [11]. However, these studies used small sample sizes of only healthy subjects. Our study included a larger sample size and used ECG-gated retinal vessel calibre measurements to examine flicker-stimulated retinal arteriolar and venular responses from baseline in diabetes mellitus compared with controls. We investigated associations between the standard digital reactive hyperaemia test (assessed by reactive hyperaemia index) and ECG-gated flicker-stimulated retinal arteriolar and venular calibres from baseline. We also explored the required sensitivity, specificity and cut-off values for reactive hyperaemia index and flicker-stimulated retinal arteriolar and venular calibres from baseline, in determining associations with diabetes mellitus.

## Methods and materials

### Study participants and ethics approval

**Fig. 1:**
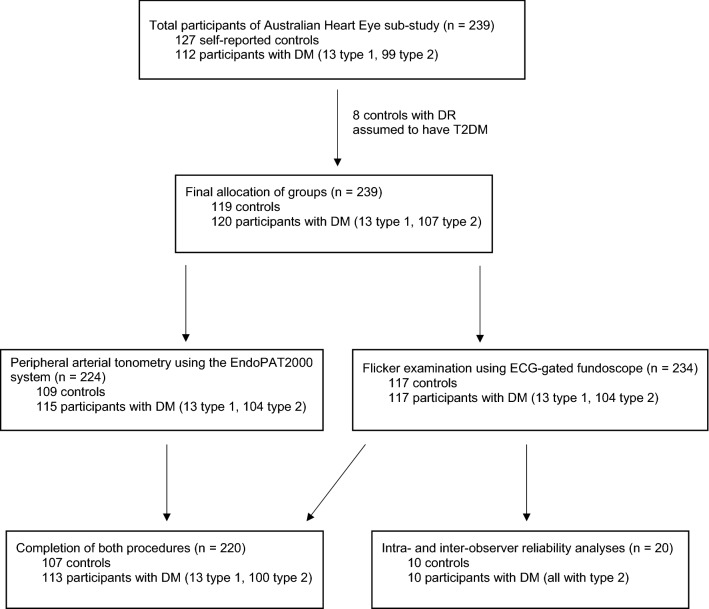
Flowchart of participants from the Australian Heart Eye sub-study who completed the non-invasive cardiovascular risk procedures (peripheral arterial tonometry and the flicker examination using the ECG-gated fundoscope) examining impaired hyperaemia. DM, Diabetes Mellitus; DR, Diabetic Retinopathy; T2DM, Type 2 Diabetes Mellitus

A total of 119 controls and 120 participants with diabetes mellitus (13 type 1, 107 type 2) were recruited for this Australian Heart Eye cross-sectional sub-study (Fig. 1) by stratified random sampling at Westmead hospital, Sydney, Australia. This method of sampling prevented selection bias. The study’s total sample size exceeded the original sample size of 50 controls and 50 patients with diabetes mellitus that was calculated to provide 80% power and a 95% level of confidence in detecting a 1% mean difference between the two groups. The study protocol was approved by the Western Sydney Local Health District Human Research Ethics Committee and followed the guidelines of the Declaration of Helsinki. Participants provided informed written consent to partake in this study, and were included regardless of pre-existing cardiovascular disease, retinal vascular occlusion, prediabetes or diabetes-related complications. The exclusion criteria included epilepsy as a precaution to flickering light exposure, elevated intraocular pressure, severe refractive error, and severe cataract.

### Data collection and anthropometric measurements

To minimise the influence of the autonomic system on peripheral macrovessels, participants were required to fast 8 hours prior to the commencement of this study including abstaining from caffeine, alcohol, smoking, food and medications. The consumption of water was permitted during the fasting period. A detailed history was collected including demographic information and a detailed medical history. Anthropometric measurements such as height (m), weight (kg) and waist circumference (cm) were measured. Body mass index (kg m^−2^) was calculated by dividing the weight by the height squared. An automated electronic device was used to measure the patient’s blood pressure (mmHg) and heart rate (bpm) (Model HEM-907; OMRON Healthcare, Victoria, Australia). Mean arterial pressure (mmHg) was calculated as follows: diastolic blood pressure + 1/3 × pulse pressure.

### EndoPAT2000 system

**Fig. 2:**
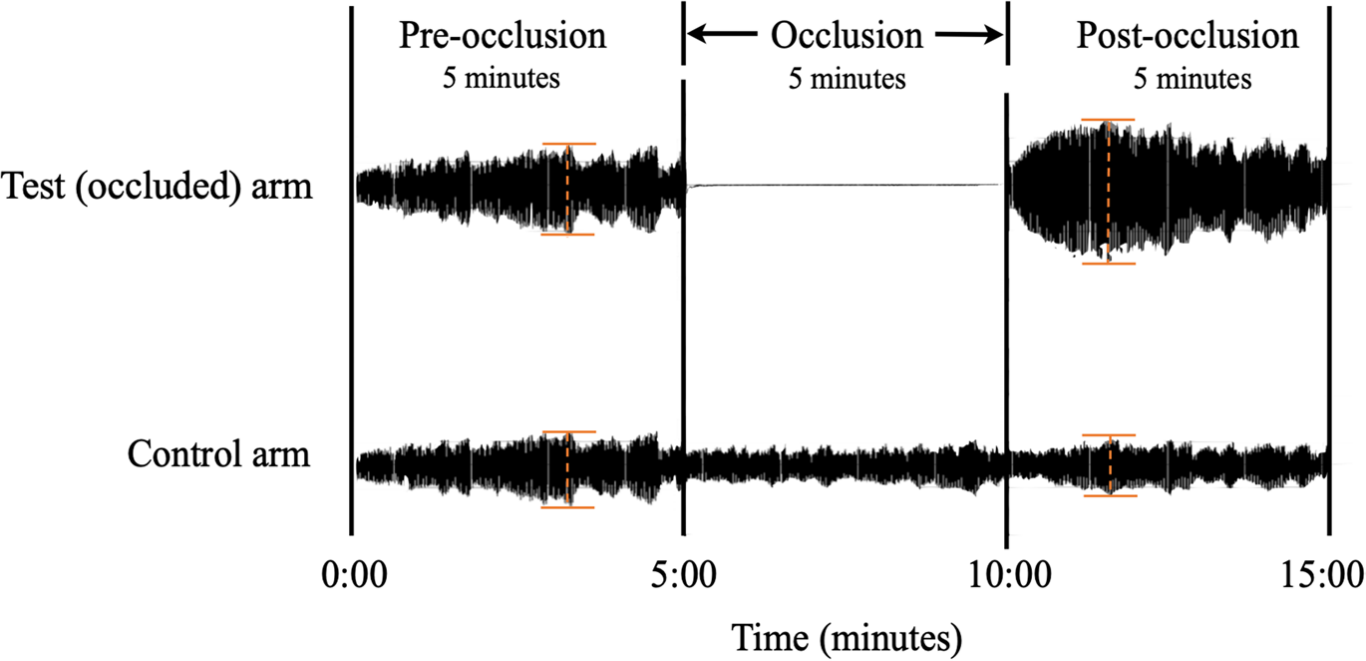
The EndoPAT2000 system’s real-time sample recording of an individual's pulsatile volume changes following brachial artery occlusion in the test arm, which the software normalises to the control arm for the calculation of reactive hyperaemia index

Patients were requested to rest by lying supinely for 5 min before the commencement of the procedure. The EndoPAT2000 device measured the pulse wave amplitude using pneumatic probes that detected the volume changes in the index finger over time. Digital reactive hyperaemia responses following occlusion of the brachial artery were examined in the test arm (non-dominant arm) of 109 controls and 115 participants with diabetes mellitus, and were normalised by measurements in the control arm (contralateral arm). The test ran for 15 minutes including a 5-min baseline, 5-min occlusion (at 220 mmHg) and 5-min post-occlusion period (Fig. 2). The EndoPAT2000 software generated reactive hyperaemia index values according to the following equation:

$${\text{Reactive hyperaemia index}} = \frac{{\text{Post to Pre-occlusion digital pulse amplitude of the test (occluded) arm}}}{{\text{Post to Pre-occlusion digital pulse amplitude of the control arm}}}$$

Reactive hyperaemia index ≤ 1.67 was the manufacturer’s recommended cut off for abnormal endothelial function and was based on a large dataset of non-selective populations [12].

### ECG-gated fundus camera with flicker stimulation

The Cardiology Research Workshop at our centre devised a retinal ECG adaptor [10] that permitted retinal images to be captured at the QRS by gating a mydriatic fundus camera (Canon CF-60DSi; Canon Inc., Tokyo, Japan) to the participant’s QRS spike. The CF-60DSi was equipped with a 21 megapixel full 35 mm frame digital camera back (EOS-1Ds Mark III; Canon Inc., Tokyo, Japan). An inhouse designed flicker module was constructed and placed in the retina illumination light path, fitted into the fundus camera's red-free filter aperture, allowing for operation with the original fundoscope design and construction. This module enabled the delivery of flickering light at 8 Hz to the participant’s eye. An average R–R was established for the participant using the last 60 R–R intervals. The ECG adaptor permitted image acquisition within a certain range from the participant’s average R–R: − 150 ms (lower limit) to 125% of the average R–R (upper limit). Image acquisition was possible after 3 consecutive beats within this acceptable range. Sinus arrhythmia, but not an ectopic beat, was within this acceptable limit. All images were acquired in RAW format (5632 × 3750 px, 14 bit).

### Retinal grading

Digital disc- and macula-centred photographs were graded for diabetic retinopathy and maculopathy according to the modified Airlie House classification of diabetic retinopathy guidelines [13] and the Wisconsin Age-related Maculopathy Grading System [14], respectively. Digital red-free, disc-centred retinal images within a 40° field of view were acquired in the left eye at 10 s intervals for a 4-min duration. The right eye of two participants with diabetes mellitus was used for grading as the left eye was unexaminable due to severe cataracts. The images included 6 photographs before flicker, 12 photographs during flicker, and 6 photographs after flicker stimulation and were used to grade retinal vessel calibre in retinal arterioles and venules. All images were de-identified in order to prevent performance bias. RetAligner (version 1.4.1), an inhouse semi-automated software, calculated retinal vessel calibre within 0.5 (900 μm) to 1 optic disc (1800 μm) diameters from the optic disc edge. The vessel width was estimated at half the peak intensity maximum using the full-width half-maximum algorithm. Retinal vessel calibre was measured in pixels and subsequently standardised to the optic disc diameter (1800 μm), which is a widely used technique [15]. The 6 preflicker, 12 flicker and 6 postflicker retinal vessel calibre measurements were each averaged. Percentage changes in retinal vessel calibre were then calculated as follows:

1. $${\text{Flicker to Preflicker}}\;(\% {\text{ change}}) = \frac{{{\text{Flicker}} - {\text{Preflicker}}}}{{{\text{Preflicker}}}} \times 100$$
2. $${\text{Postflicker to Flicker}}\;(\% {\text{ change}}) = \frac{{{\text{Postflicker}} - {\text{Flicker}}}}{{{\text{Flicker}}}} \times 100$$
3. $${\text{Postflicker to Preflicker}}\;(\% {\text{ change}}) = \frac{{{\text{Postflicker}} - {\text{Preflicker}}}}{{{\text{Preflicker}}}} \times 100$$

In a subset of 10 controls and 10 participants with diabetes, the same reviewer measured retinal vessel calibre in the same retinal arterioles and venules but using different slice sections of the vessels. In the same subset of participants, a second reviewer also measured the retinal vessel calibre in the same retinal arterioles and venules but using different slice sections than reviewer 1.

### Statistics

Raw retinal vessel calibre data were transferred from the RetAligner software into Excel for conversion to microns, and averages calculated at the three intervals (preflicker, flicker and postflicker) of the study. All data were entered onto SPSS for Macintosh version 26.0 (SPSS Inc., Chicago, IL, USA) for statistical analyses. SPSS and Prism version 9.2.0 for macOS (GraphPad Software Inc., San Diego, CA, USA) were used to generate the graphs. Shapiro–Wilk test was used to test the data for normality. Valid nominal data were organised as mean, standard deviation (SD) and 95% confidence interval (95%CI), or as median and interquartile range (IQR). Valid categorical data were represented as frequency and percentages. Pearson’s *χ*^2^ test compared categorical data between groups. In groups with multiple comparisons, this was followed by a Bonferroni *χ*^2^ residual analysis to identify significant pairs. Paired samples *t* tests compared the mean of nominal variables within groups and the independent student’s *t* test between groups. Independent Mann–Whitney *U* test compared the median of nominal variables between groups. Spearman's rho correlation coefficient assessed statistically significant strength of associations of nominal variables. The multiple linear stepwise regression model was used to investigate associations between flicker-stimulated arteriolar calibre from baseline with reactive hyperaemia index and flicker-stimulated retinal venular calibre from baseline. The model was built on the variables age, sex, body mass index, waist circumference, ethnicity, diabetes mellitus, retinopathy, hypertension, hypercholesterolaemia, fatty liver, coronary heart disease, peripheral vascular disease, smoking status, mean arterial pressure, systolic blood pressure, diastolic blood pressure, heart rate, reactive hyperaemia index, and flicker-stimulated retinal arteriolar and venular calibre changes from baseline. A receiver operating characteristic curve determined the associations of reactive hyperaemia index, flicker-stimulated retinal venular calibre from baseline with diabetes mellitus. The interclass correlation coefficient was used for intra- and inter-observer reliability analyses in 10 controls and 10 participants with diabetes mellitus. Bonferroni correction was applied to an alpha level of 0.05 and therefore *p* values < 0.0167 (0.05/3 comparisons) were considered statistically significant, unless otherwise indicated.

## Results

### Baseline characteristics of study participants

Of all study participants, 91.6% completed both procedures (Fig. 1). The main reason for not completing both procedures was time constraints. Table 1 summarises the participant characteristics of the study. Compared with controls, participants with diabetes mellitus had a higher mean age, body mass index, waist circumference, mean arterial pressure, systolic blood pressure, diastolic blood pressure, and heart rate. Caucasians and South Asians formed a large proportion of participants. A greater proportion of participants with diabetes mellitus were ex-smokers, and a lower proportion had never smoked, compared with controls. A higher proportion of participants with diabetes mellitus had hypertension, hypercholesterolaemia and fatty liver (1.8% controls vs 14.3% diabetes mellitus, *p* < 0.01) compared to controls. All participants’ health conditions were well controlled with medications. Fundoscopic grading revealed that 32.5% of participants with diabetes mellitus had established diabetic retinopathy.

**Table 1:**
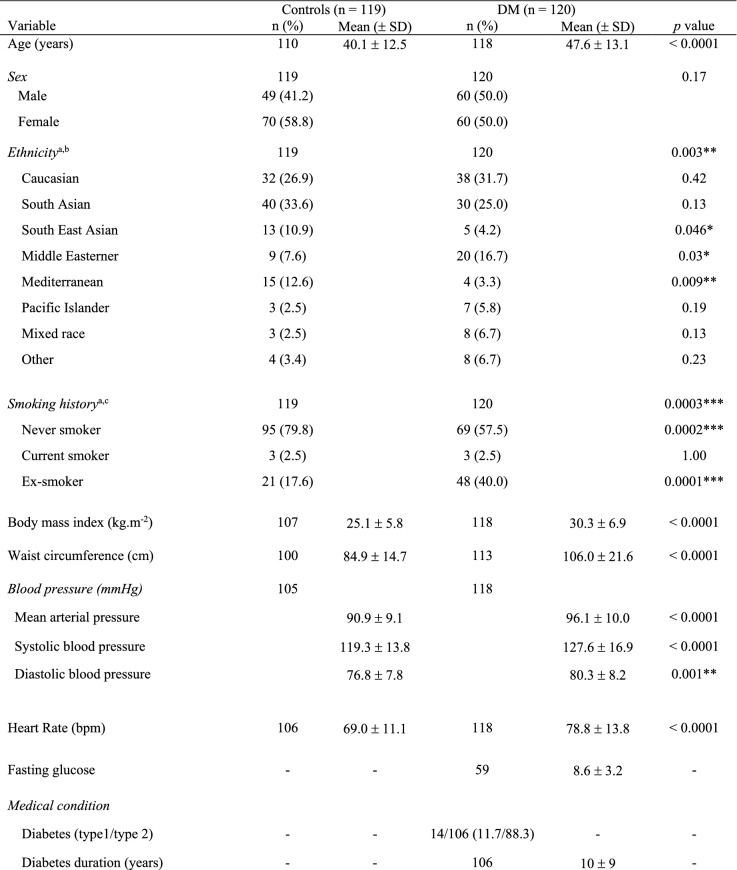

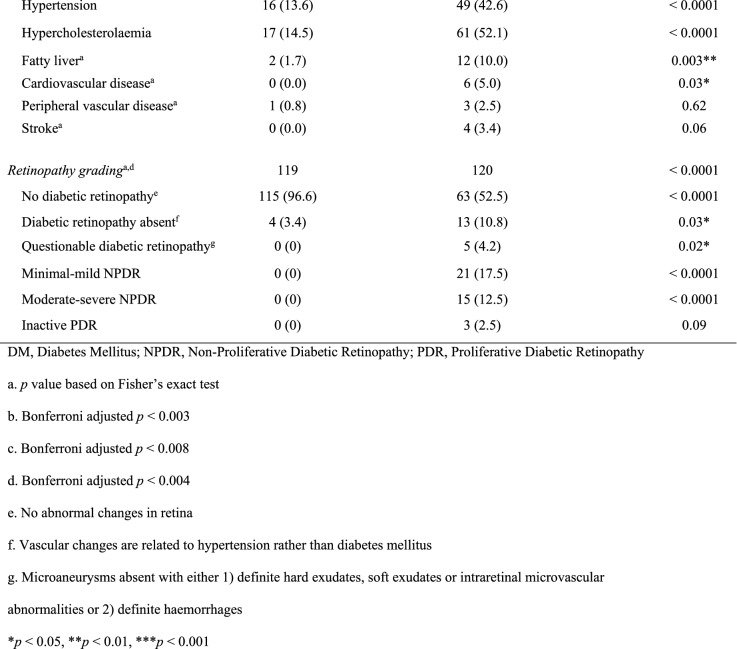
Participant baseline characteristics

DM, diabetes mellitus; NPDR, non-proliferative diabetic retinopathy; PDR, proliferative diabetic retinopathy

^*^Bonferroni-corrected *p* < 0.003

^†^Bonferroni-corrected *p* < 0.004

^‡^Bonferroni-corrected *p* < 0.008

### Reactive hyperaemia index measurements in participants with and without diabetes mellitus

**Fig. 3:**
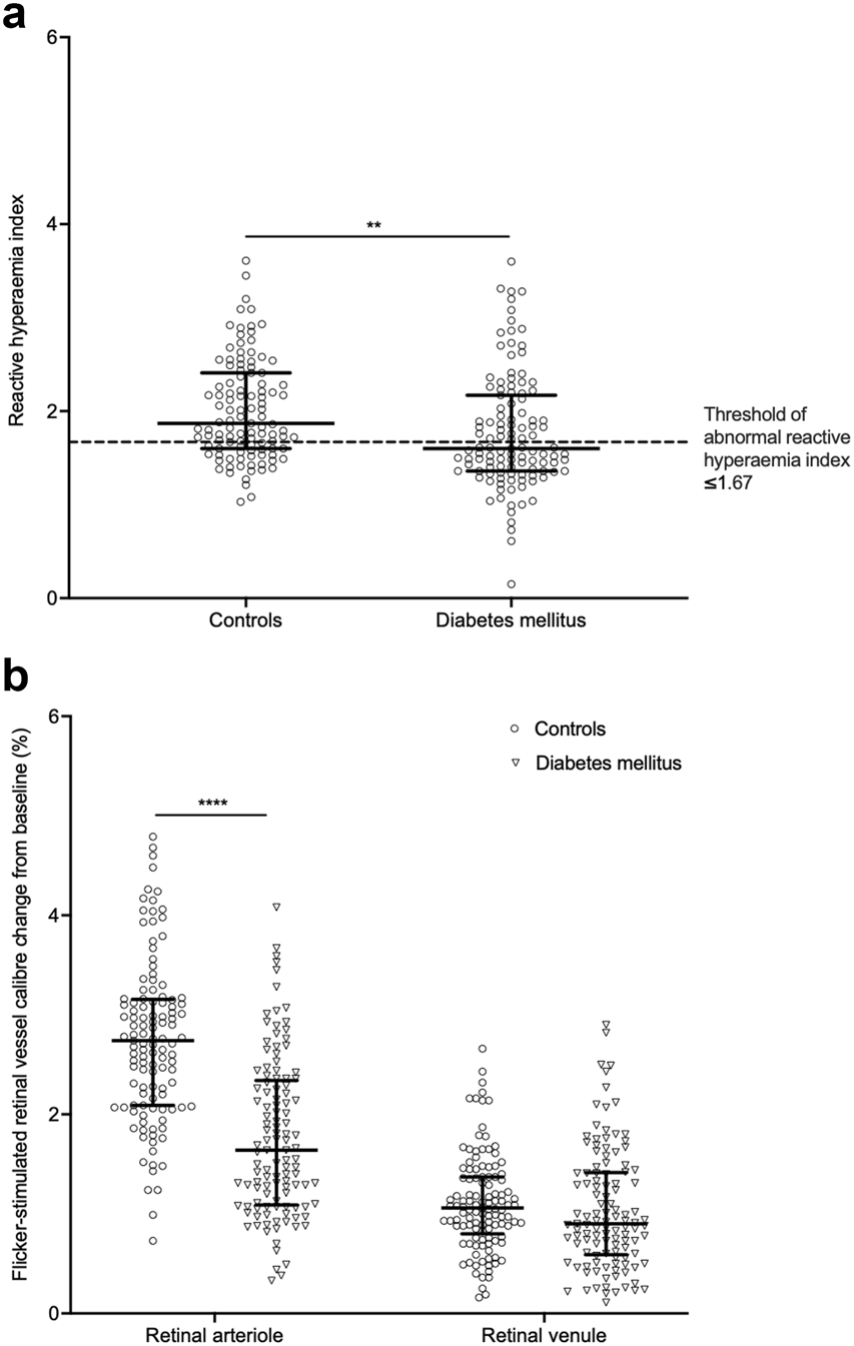
A comparison between controls and diabetes mellitus of **a** median reactive hyperaemia index and **b** median flicker-stimulated retinal arteriolar and venular calibre changes from baseline. Error bar represents IQR. **p < 0.01; ****p < 0.0001

Of the 109 controls and 115 participants with diabetes mellitus in this analysis, median reactive hyperaemia index was lower in diabetes mellitus compared with controls (controls: 1.87, IQR 0.81 vs diabetes mellitus: 1.60, IQR 0.81, *p* = 0.0003). Figure 3a shows an abnormal reactive hyperaemia index ≤ 1.67 was observed in 31.2% of controls and 53.9% of participants with diabetes mellitus.

### Flicker-stimulated retinal arteriolar and venular calibre changes from baseline in participants with and without diabetes mellitus

Of the 117 controls and 117 participants with diabetes mellitus included in the analysis, flicker-stimulated retinal arteriolar and venular calibre changes were significantly greater than baseline in controls and participants with diabetes mellitus (*p* < 0.0001). Fig. 3b shows the median flicker-stimulated retinal arteriolar calibre change from baseline was lower in diabetes mellitus than controls (controls: 2.74%, IQR 1.07 vs diabetes mellitus: 1.64%, IQR 1.25, *p* < 0.0001) and the median flicker-stimulated retinal venular calibre change from baseline was similar between the two groups (controls: 1.06%, IQR 0.57 vs diabetes: 0.90%, IQR 0.83, *p* = 0.09).

Of the 40 participants with diabetic retinopathy and 77 participants without diabetic retinopathy, no statistically significant difference occurred in flicker-stimulated retinal arteriolar calibre change from baseline in participants with diabetes mellitus who had diabetic retinopathy compared with those without diabetic retinopathy (1.59% vs 1.85% respectively, *p* = 0.10). There was also no statistically significant difference in flicker-stimulated retinal venular calibre change from baseline between participants with diabetes mellitus who had diabetic retinopathy compared with those without diabetic retinopathy (0.93% vs 1.10% respectively, *p* = 0.18).

**Fig. 4:**
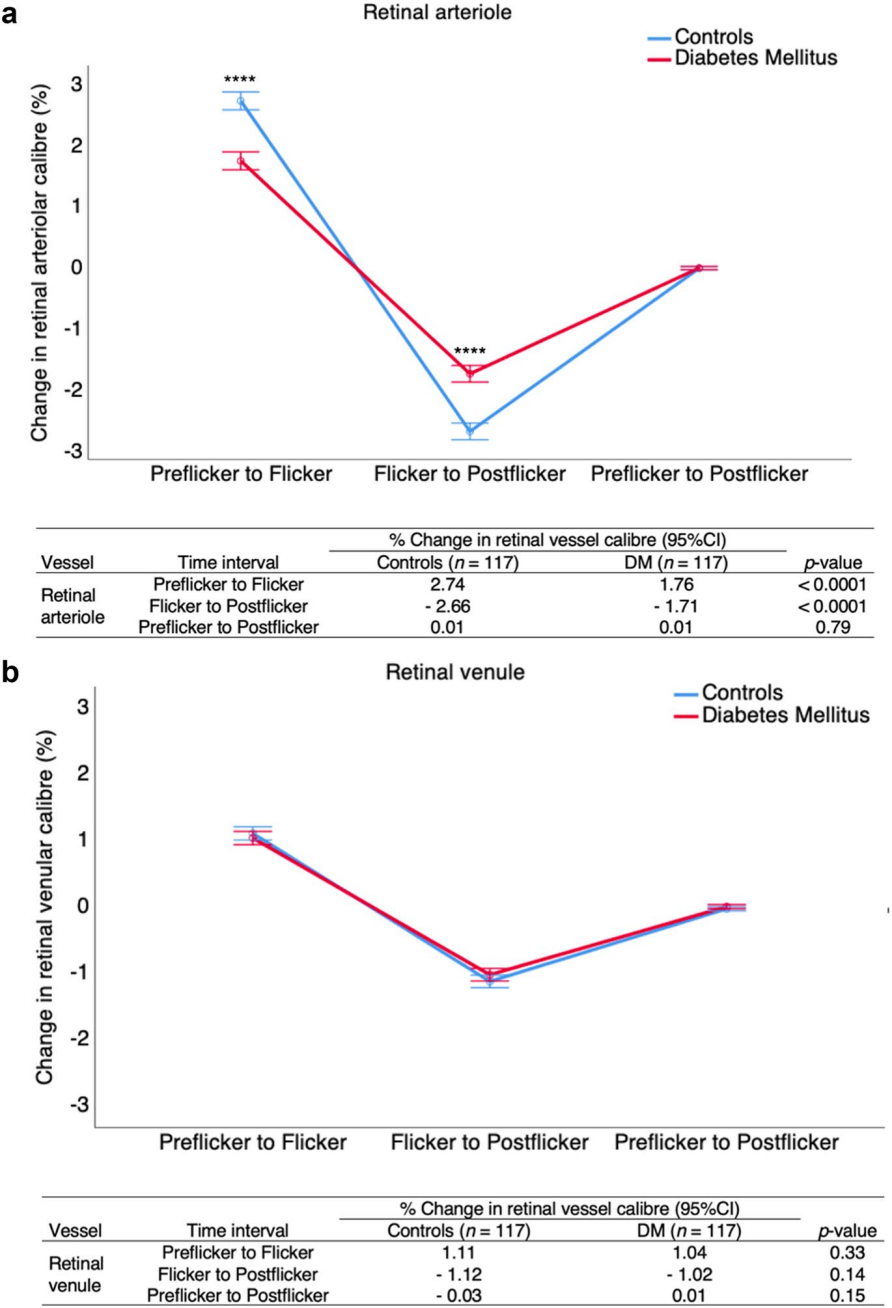
The degree of retinal arteriolar and venular calibre changes between different periods of the ECG-gated retinal examination. Error bar represents 95% CI of the mean. DM, diabetes mellitus. ****p < 0.0001

Figure 4 shows that in participants with diabetes mellitus, there was a smaller increase in retinal arteriolar calibre from preflicker to flicker and a smaller decrease in retinal arteriolar calibre from flicker to postflicker, compared with controls. No statistically significant change between the two groups occurred in retinal arteriolar calibre from preflicker to postflicker. In retinal venules, there was no statistically significant difference between the two groups in retinal venular calibre from preflicker to flicker, flicker to postflicker, and preflicker to postflicker.

### Correlation of reactive hyperaemia index with flicker-stimulated retinal arteriolar and venular calibre changes from baseline

Flicker-stimulated arteriolar calibre change from baseline was weakly correlated with reactive hyperaemia index (*r* = 0.19, *p* = 0.005) and flicker-stimulated retinal venular calibre change from baseline (*r* = 0.27, *p* < 0.0001). Flicker-stimulated retinal venular calibre change from baseline was not correlated with reactive hyperaemia index (*r* = 0.08, *p* = 0.26).

### Multiple linear stepwise regression

A Bonferroni corrected  *p *< 0.0026 (0.05/19 predictor variables) was attributed statistical significance. After adjusting for age and smoking status, flicker-stimulated retinal arteriolar calibre change from baseline was associated with diabetes mellitus (*β* =  − 0.80, *p* < 0.0001) and flicker-stimulated venular calibre change from baseline (*β* = 0.34, *p* = 0.0008), but this association did not reach statistical significance with reactive hyperaemia index (*β* = 0.22, p = 0.02). Reactive hyperaemia index demonstrated a statistically significant association with diabetes mellitus only (*β* =  − 0.27, *p* = 0.001).

### Intra- and inter-observer repeatability of flicker-stimulated retinal arteriolar and venular calibre changes from baseline

The intra-observer repeatability was high in retinal arteriolar (interclass correlation coefficient 0.93, 95% CI 0.84–0.97, *p* < 0.0001) and venular (interclass correlation coefficient 0.87, 95% CI 0.71–0.95, *p* < 0.0001) calibre changes from baseline. The inter-observer repeatability was also high in retinal arteriolar (interclass correlation coefficient 0.93, 95% CI 0.82–0.97, *p* < 0.0001) and venular (interclass correlation coefficient 0.90, 95% CI 0.74–0.96, *p* < 0.0001) calibre changes from baseline. This shows that there was good agreement of measurements within the same reviewer and between reviewers.

### Receiver operating characteristic curve for reactive hyperaemia index and flicker-stimulated retinal arteriolar and venular calibre changes from baseline

**Fig. 5:**
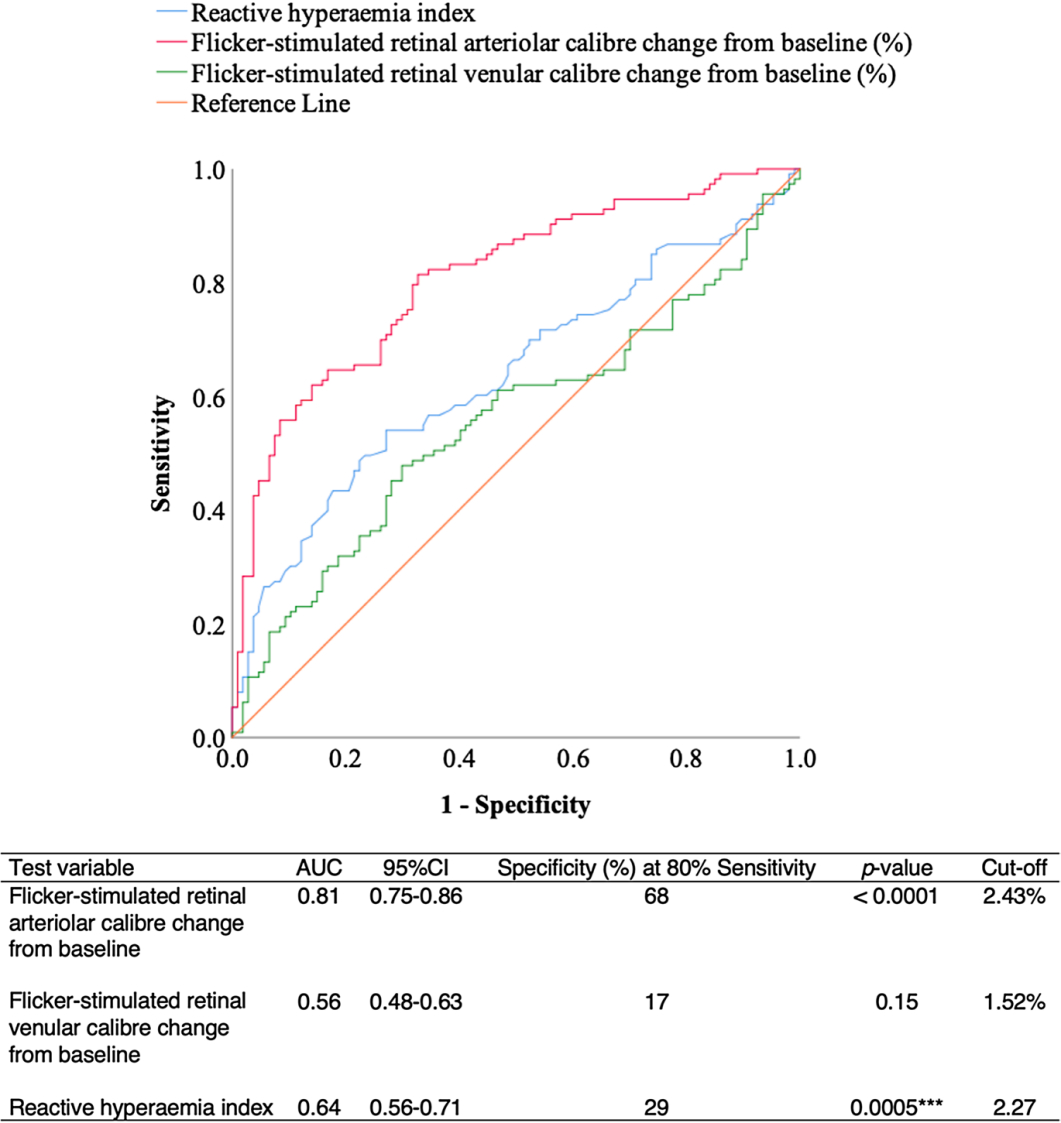
Receiver operating characteristic curve comparing associations of diabetes mellitus with flicker-stimulated retinal arteriolar and venular calibre changes from baseline, and peripheral artery tonometry. AUC, area under the curve. ***p < 0.001

Figure 5 shows that of the total study participants, 107 controls and 113 participants with diabetes mellitus were examined by both procedures where flicker-stimulated retinal arteriolar calibre change from baseline was strongly associated with diabetes mellitus and had a good specificity at 80% sensitivity. Reactive hyperaemia index and flicker-stimulated retinal venular calibre change from baseline were poorly associated with diabetes mellitus and had low specificities at 80% sensitivity. At the manufacturer’s recommended reactive hyperaemia index cut-off value of 1.67, the sensitivity was poor (54%) and the specificity was good (69%).

## Discussion

The aim of this study was to assess the flicker-stimulated hyperaemia response in diabetes mellitus compared with controls by single retinal calibre measurements obtained from static retinal images using our novel ECG-gated fundoscope. These measurements were compared to the commonly used method of peripheral artery tonometry (reactive hyperaemia index). Flicker-stimulated retinal arteriolar calibre change from baseline and reactive hyperaemia index were reduced in diabetes mellitus compared to controls. Flicker-stimulated retinal venular calibre change from baseline was similar in both groups. We also observed a weak association between reactive hyperaemia index and flicker-stimulated retinal arteriolar calibre change from baseline. The weak association may have been due to the different provocation techniques used (post-brachial ischaemic hyperaemia vs flicker stimulation), and the distinct biomechanical properties of the different vascular beds (large arteries vs retinal microvessels). As expected, flicker-stimulated retinal arteriolar calibre from baseline was positively correlated with flicker-stimulated retinal venular calibre from baseline, since people with greater arteriolar dilation responses tended to have greater venular dilation responses [16].

Our results were in agreement with the expected finding of a reduced reactive hyperaemia index in diabetes mellitus [17, 18]. However, this was contrary to other studies that observed no differences between controls and diabetes mellitus [19–21]. The discrepancies between studies could be explained by autonomic innervation influencing peripheral vasomotor tone in larger peripheral vessels [22]. In our study, the Framingham-corrected reactive hyperaemia index had a higher correlation with diabetes mellitus than reactive hyperaemia index, and appears to be a more suitable marker for assessing endothelial function in populations susceptible to cardiovascular risk factors [23]. The usefulness of the EndoPAT is therefore questionable as the traditional Framingham risk score, a cheaper method than the expensive EndoPAT system, has better sensitivity for endothelial function in diabetes mellitus.

To date, various studies have used real time flicker-stimulated responses of retinal vessels, by devices such as the Dynamic Vessel Analyzer, to examine endothelial function. The general consensus in these studies was that in diabetes mellitus, flicker-stimulated responses were impaired in both retinal arterioles and venules, with only one study showing no impairment in either vessel [19]. Our study supports the presence of underlying endothelial dysfunction within the retinal arteriolar microcirculation of people with diabetes mellitus. We observed that flicker-stimulated retinal arteriolar calibre from baseline was reduced in diabetes mellitus compared to controls, but unlike previous studies, no statistically significance difference in flicker-stimulated retinal venular calibre from baseline occurred between the two groups. These studies used average measurements across a minimum of six retinal arterioles and venules (central retinal arteriolar and venular equivalents), which are known to mask dilatory capacity and reaction patterns of individual vessels [24], compared to single retinal vessel calibre measurements employed in our study. The Dynamic Vessel Analyzer camera used in these studies also had a lower pixel resolution than the ECG-gated photographs captured in our study (720 × 575 px (PAL standard) vs 5632 × 3750 px), making it more difficult to detect small retinal vascular responses. This device also exposed participant eyes to 12.5 Hz of flickering light, compared to 8 Hz in our study that we based on previous studies [25, 26]. We did not use 12.5 Hz of flickering light as it is a submultiple of 50 Hz - a commonly used frequency in fluorescent lights. Therefore, by using an 8 Hz frequency, we avoided the superposition of two similar flicker frequencies, and prevented light to flicker at an undesired frequency.

The reduced flicker-stimulated retinal arteriolar calibre change from baseline in diabetes mellitus may be related to early pathological microvascular changes, such as wider arteriolar calibres observed in many large population-based studies [27–30]. Some population-based studies also found associations of wider venular calibre with diabetes mellitus, but this was in relation to the degree of diabetic retinopathy or increased disease duration [24, 31] and was not present with incident diabetes [32]. A wider venular calibre indicated a predilated state and was thought to explain the reduced flicker-stimulated retinal venular calibre change from baseline in diabetes mellitus because of the reduced residual dilatory capacity [33]. Since our study demonstrated no association of diabetic retinopathy status with retinal vascular response, and many participants were newly diagnosed with diabetes mellitus, it may explain why the flicker-stimulated retinal venular calibre change from baseline was similar in the two groups.Veins are thin walled and are usually compliant to volume changes rather than active contraction. As such, it is expected that the flicker-stimulated retinal venular calibre from baseline is unchanged. 

Finally, we found that a reduced flicker-stimulated retinal arteriolar calibre change from baseline had good sensitivity and specificity for diabetes mellitus, whereas reduced reactive hyperaemia index and flicker-stimulated retinal venular calibre change from baseline had poor specificities. This is because retinal arteriolar endothelial dysfunction occurs early in diabetes mellitus [34]. Retinal arteriolar endothelial dysfunction may even precede the diagnosis of diabetes mellitus [35], and therefore any macrovascular and venular microvascular changes [36]. However, this remains to be proven. A similar finding was observed in a prospective study where impaired retinal arteriolar endothelial function was sensitive in detecting the presence of coronary artery disease, but the responses from digital reactive hyperaemia or flow-mediated dilation were unable to differentiate between people with and without coronary artery disease [37]. Our results suggest that a reduced flicker-stimulated arteriolar calibre change from baseline has a good ability to correctly categorise people with pre-existing diabetes mellitus. However, this conclusion is limited to the participants of this study. Future studies are required to explore the applicability of this conclusion on a larger population-based level.

This study’s main limitation was the inability to determine the true correlation between endothelial function in diabetes mellitus and flicker-stimulated retinal arteriolar calibre change from baseline. To establish the true endothelial function in all participants, a comparison of our data with the invasive gold standard of catheterisation is required. In order to determine how early the flicker-stimulated changes happen during the pathogenesis of diabetes mellitus, a prospective cohort study in the general population free of comorbidities but with risk factors for diabetes mellitus (e.g. family history of diabetes mellitus, high body mass index, abdominal obesity, ethnicity) or with pre-diabetes would need to be employed and monitored over time with fasting glucose levels, oral glucose tolerance tests (if appropriate) and flicker examinations. This will enable identifying those who develop diabetes mellitus, tracking their progression, and determining how early the flicker-stimulated changes occur in the pathogenesis of diabetes mellitus.

Another limitation of the study was the relatively small sample size. However, the sample consisted of a large variety of ethnicities representative of the Australian population, thereby enhancing its external validity at a national level and to other culturally diverse countries. Larger population-based studies are still required to confirm our findings. The direction of causality of flicker-stimulated retinal vascular reactivity could also not be established with reactive hyperaemia index and diabetes mellitus due to the cross-sectional design of this study. The static nature of fundus images did not allow temporal measurements of retinal blood flow. This could have been overcome by repeating the cycles of flickering light administered or by repeating the same test on a preceding day. Also, improving the current design of the fundoscope could increase participant comfort and compliance throughout retinal examinations [38], which is important for enhancing the image quality captured for retinal vessel analyses.

Our study also had some major strengths, including it being the first study to determine the correlation of the cardiovascular risk factors, impaired digital reactive hyperaemia and flicker-stimulated retinal vascular response, with diabetes mellitus. This study was sufficiently powered to detect a small but clinically significant impairment of microvascular response in a greater proportion of diabetes mellitus compared with peripheral artery tonometry. Hence, the ECG-gated fundoscope may improve the detection of abnormal vascular changes in the pathogenesis of diabetes mellitus, which is clinically important in the secondary prevention of diabetes-related complications. We also reported high intra- and inter-observer correlations of flicker-stimulated retinal arteriolar and venular calibre changes from baseline, using randomly selected slice points, in the same retinal vessels, demonstrating that this method is repeatable.

Overall, an ECG-gated flicker-stimulated retinal arteriolar calibre change from baseline was more strongly correlated with diabetes mellitus than peripheral artery tonometry. The relationship between an impaired ECG-gated flicker-stimulated retinal arteriolar calibre change from baseline and endothelial dysfunction, in diabetes mellitus, remains to be elucidated.

## Data Availability

The raw quantitative dataset used to support the findings of this study is deidentified participant data and is available from the corresponding author on reasonable request. Please contact Dr. Anchal Lal: alal2824@alumni.sydney.edu.au if interested.
